# Focus on the Predictive Value of Subclassification of Extratumoral Structural Abnormalities for Malignant Nonspiculate and Noncalcified Masses on Digital Mammography

**DOI:** 10.3389/fgene.2022.822858

**Published:** 2022-02-04

**Authors:** Ye Xu, Jianghong Sun, Fei Guo, Abiyasi Nanding, Qiyang Li, Dan Jiang

**Affiliations:** ^1^ Department of Radiology, Harbin Medical University Cancer Hospital, Harbin, China; ^2^ Department of Pathology, Harbin Medical University Cancer Hospital, Harbin, China

**Keywords:** breast, nonspiculate and noncalcified masses, extratumoral structural abnormalities, predictive value, digital mammography

## Abstract

**Purpose:** To determine the independent risk factors associated with malignant nonspiculate and noncalcified masses (NSNCMs) and evaluate the predictive values of extratumoral structural abnormalities on digital mammography.

**Methods:** A total of 435 patients were included between January and May 2018. Tumor signs included shape, density, and margin, which were evaluated. Extratumoral signs were classified into extratumoral structural abnormalities (parenchymal and trabecular) and halo; subclassification included contraction, distortion, pushing and atrophy sign of parenchyma, parallel, vertical, and reticular trabecula sign, and narrow and wide halo. Univariate and multivariate analysis was performed. The positive predictive value (PPV) of the independent predictor was calculated, and diagnostic performance was evaluated using the receiver operating characteristic curve.

**Results:** Of all cases, 243 (55.8%) were benign and 192 (44.2%) were malignant. Extratumoral contraction sign of parenchyma was the strongest independent predictor of malignancy (odds ratio [OR] 36.2, *p* < 0.001; PPV = 96.6%), followed by parenchymal distortion sign (OR 10.2, *p* < 0.001; PPV = 92%), parallel trabecula sign (OR 7.2, *p* < 0.001; PPV = 85.6%), and indistinct margin of tumor (OR 4.3, *p* < 0.001; PPV =70.9%), and also parenchymal atrophy sign, wide halo, vertical trabecula, age ≥ 47.5 years, irregular shape, and size ≥ 22.5 mm of tumor (OR range, 1.3-4.0; PPV range, 56.6-83.6%). The diagnostic performance of most of the extratumoral signs was between that of indistinct margin and irregular shape of tumor.

**Conclusion:** The subclassification of extratumoral structural abnormalities has important predictive value for mammographic malignant NSNCM, which should be given more attention.

## Introduction

Breast cancer remains a global public health problem (Masood and Rosa, 2011). The incidence of breast cancer in Chinese women continues to rise (Bai et al., 2020). Digital mammography is one of the important imaging tools for breast cancer screening and diagnosis (Fischer et al., 2006; Zeeshan et al., 2018). The morphological analysis of mammographic signs is still one of the research tasks of radiologists, regardless of the development of imaging technology and artificial intelligence.

Mass is the most common imaging manifestation of breast cancer and also the main sign of benign disease. Digital mammography descriptors include shape, density, and margin according to the Breast Imaging Data and Reporting System (BI-RADS), which are further classified in detail (Fischer et al., 2006; Zeeshan et al., 2018). Spiculate mass is more likely to be evaluated as malignancy because of its very high positive predictive value (Burrell et al., 1996; Liberman et al., 1998). Calcifications may be associated with mass, and the type of calcification will increase radiologists’ confidence in evaluating mass. Then, we classified the remaining masses as nonspiculate and noncalcified masses (NSNCMs). We are interested in these types of masses because more attention is often required to consider malignant possibility.

A review of previous literatures related to mammographic masses showed that most of them focused on signs of the mass itself. The most common impression is that round or oval mass with circumscribed margin is more likely to be benign, whereas a malignant mass has irregular shape (Liberman et al., 1998; Berment et al., 2014; Li et al., 2017; Nakashima et al., 2017; Woods et al., 2021). However, some malignant tumors also present circumscribed margin (Meyer et al., 1989; Wang et al., 2008; Yoo et al., 2010). Therefore, more morphologic information should be mined to predict malignant NSNCMs on mammography and provide clues for clinical management decisions.

The interaction between the tumor and the microenvironment is an important mechanism in the process of tumor growth and metastasis (Troester et al., 2009). The evolution of breast cancer requires co-optation of the surrounding stromal tissues to facilitate progression and support metabolic demand (Jones et al., 2013). Normal-appearing stromal tissues surrounding breast tumors can harbor abnormalities (Li et al., 2002). Therefore, our study will explore the classification and subclassification of extratumoral signs, which was rarely seen in the previous literature. The purpose of this study is to determine the independent risk factors associated with malignant NSNCMs and evaluate the predictive value of subclassification of extratumoral structural abnormalities by analyzing tumor signs and extratumoral signs on digital mammography.

## Materials and Methods

### Patients

The hospital institutional review board approved our observational study and waived the need for informed consent because the study was performed retrospectively using routinely acquired mammograms.

**FIGURE 1:**
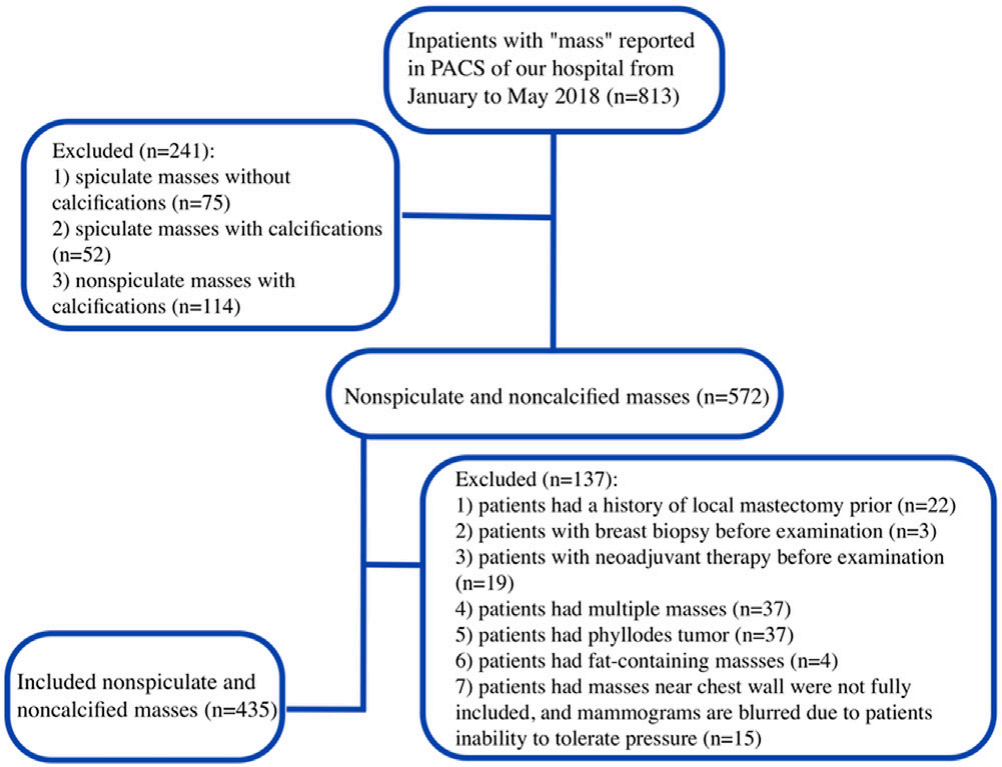
Flowchart shows the process of enrolling patients in this study.

The keyword “mass” was searched in the digital mammography report interface of the picture archiving and communication system (PACS) of our hospital. The limited date was from January to May 2018, and the subject was inpatients. There were 813 patients in total; subsequently, there were 241 (29.6%) spiculate masses and calcified masses, and 137 (16.9%) cases not suitable for this study were excluded (Figure 1) Eventually, 435 (53.5%) patients with NSNCMs were included, who underwent surgery and were pathologically confirmed. They were all female, and the mean age ±standard deviation was 46.1 ± 11.6 years (range, 16–76 years).

### Digital Mammography

Mammograms were obtained using a full field digital mammography system (MS-3500, Fuji, Japan; Inspiration, Siemens, Germany). Examinations were performed by experienced technologists using the automatic exposure mode, and the manual exposure mode was used when the mass was large. Conventional craniocaudal and mediolateral oblique views were obtained. The supplemented position was performed when necessary. The examinational pressure was based on the maximum tolerance of the patient by communication. All mammograms were transmitted to both the picture archiving and communication system and the diagnosis workstation.

### Imaging Analysis

Each enrolled patient had 4 images [2 in mediolateral oblique (MLO) view and 2 in craniocaudal (CC) view]. The MLO view image size was 65.67*82.33 inches, and the resolution was 300 dpi. The CC view image size was 28.92*38.89 inches, and the resolution was 300 dpi.

Two radiologists, respectively, reviewed all mammographic views on the specialized diagnostic workstation (5.8 M dual display screen) without knowledge of the pathological diagnosis. The imaging data were recorded by a radiologist with 5 years of breast imaging experience and reviewed by a deputy chief physician who has been engaged in mammography diagnosis for 16 years. Both two radiologists reached a consensus after discussion for inconsistent descriptors.

Tumor signs were evaluated and recorded using the BI-RADS lexicon (Uchiyama and Fukuda, 1989; Zhou et al., 2014), including shape (round, oval, and irregular), density (high, equal, and low), and margin (circumscribed, obscured, and indistinct). At the same time, the mass size was recorded and based on its largest diameter.

**FIGURE 2:**
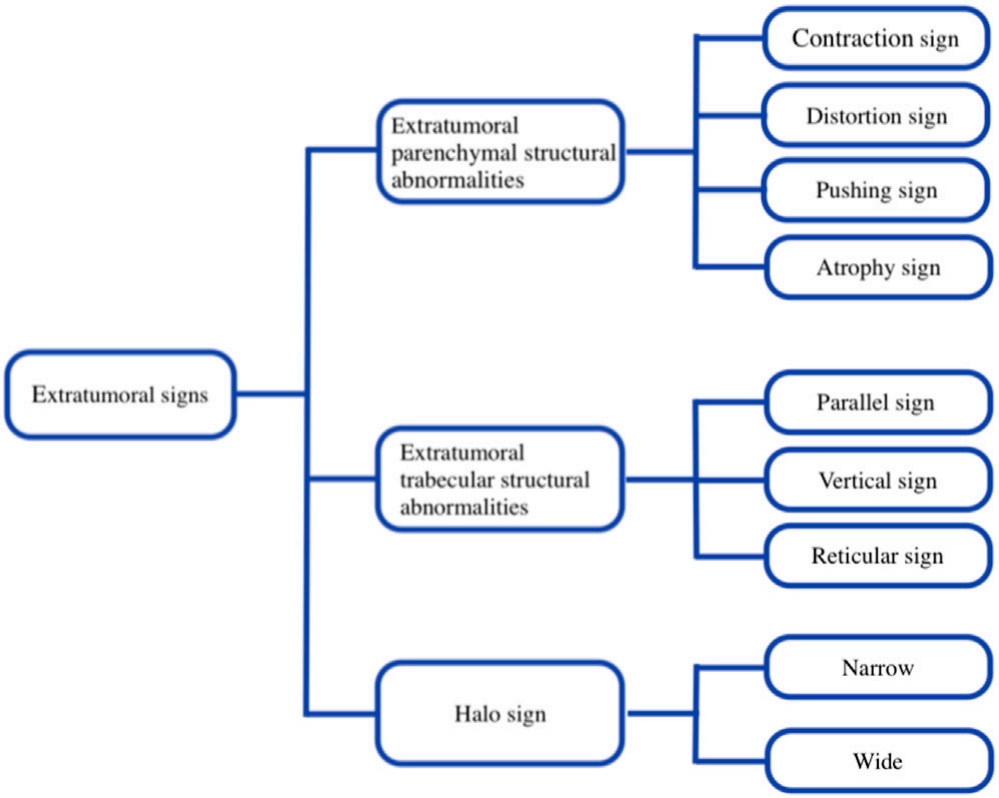
Flowchart shows the subclassification of extratumoral signs in this study.

**FIGURE 3:**
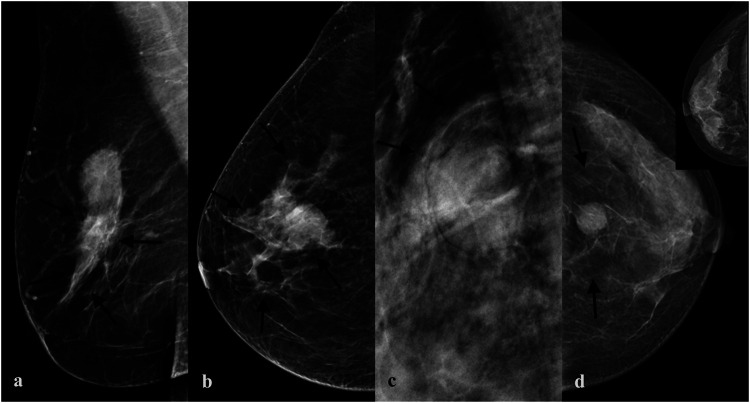
Subclassification of parenchymal abnormalities. **(A)** A mass with extratumoral contraction sign of parenchyma in a 49-year-old woman proved to be invasive ductal carcinoma grade Ⅱ pathologically. **(B)** A mass with extratumoral distortion sign of parenchyma in a 48-year-old woman proved to be invasive ductal carcinoma grade Ⅲ pathologically. **(C)** A mass with extratumoral pushing sign of parenchyma in a 45-year-old woman proved to be fibroadenoma pathologically. **(D)** A mass with extratumoral atrophy sign of parenchyma in a 45-year-old woman proved to be invasive ductal carcinoma grade Ⅲ pathologically.

**FIGURE 4:**
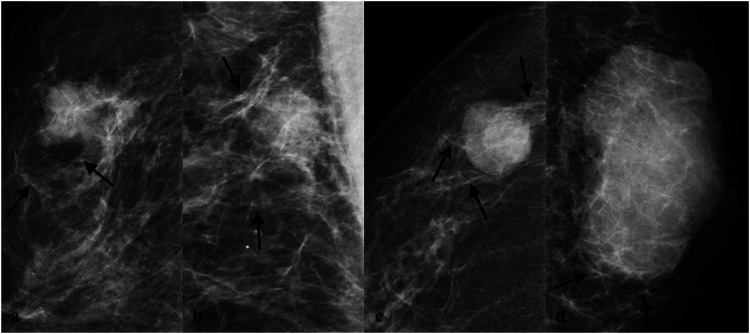
Subclassification of trabecular abnormalities. **(A)** A mass with parallel trabecula sign in a 56-year-old woman proved to be invasive ductal carcinoma grade Ⅲ pathologically. **(B)** A mass with parallel trabecula sign in a 55-year-old woman proved to be invasive ductal carcinoma grade Ⅲ pathologically. **(C)** A mass with vertical trabecula sign in a 61-year-old woman proved to be invasive ductal carcinoma grade Ⅲ pathologically. **(D)** A mass with reticular trabecula sign in a 62-year-old woman proved to be invasive ductal carcinoma grade Ⅱ pathologically.

In this study, based on BI-RADS and our breast imaging experience, extratumoral signs of NSNCM were classified into extratumoral parenchymal structural abnormalities, extratumoral trabecular structural abnormalities, and halo signs, which were further subclassified (Figure 2). The subclassification of parenchymal abnormalities included contraction, distortion, pushing, and atrophy sign (Figure 3). The detailed explanation was as follows: parenchymal contraction sign was defined as aggregation and contraction toward the mass, distortion sign was described as losing normal texture, pushing sign meant that the displacement of parenchyma due to compression of mass, and atrophy sign meant the reduction of parenchyma outside the mass compared to the normal contralateral area. Then, the trabecular abnormalities were subclassified according to the direction to the edge of the mass, including parallel, vertical, and reticular trabecula signs (Figure 4). Halo signs were also divided into narrow (width <0.5 mm) and wide (width ≥0.5 mm). All of the extratumoral signs were detected in at least one mammographic view.

### Statistical Analysis

Continuous data were expressed as mean ± standard deviation, and categorical variables were expressed as a percentage. First, univariate analysis was performed, and the Student’s *t*-test was used for identifying the differences of age and mass size between the two groups. Tumor signs and extratumoral signs between benign and malignant NSNCMs were compared using the chi-square test (with Yates correction) and Fisher’s exact test. Multivariate logistic regression analysis was subsequently performed to determine independent risk factors for malignancy. All variables with *p* < 0.2 at univariate analysis were considered for the multivariate model (David and Hosmer, 2013). At the same time, optimal cut-off values of age and mass size for distinguishing malignant from benign were estimated by a receiver operating characteristic (ROC) curve analysis (Youden index), which were also taken into multivariate regression analysis. Here, we adopted the stepwise method to select variables and obtained the independent risk factors of malignant NSNCM.

The positive predictive value (PPV), negative predictive value (NPV), sensitivity, and specificity of independent risk factors were calculated using histopathological diagnosis as the standard of reference. The diagnostic performance for the significant independent predictor was estimated as the area under the receiver operating characteristic curve (AUC). The diagnostic performance was regarded as low (AUC = 0.5–0.6), moderate (AUC = 0.6–0.8), or high (AUC >0.8) (Xu et al., 2014).

All statistical analyses were performed by using R software (version 4.0.3; R Development Core Team, Vienna, Austria). A level of *p* < 0.05 was considered to indicate a significant difference.

## Results

### Pathologic Diagnosis

Of all 435 NSNCMs, 243 (55.8%) were benign and 192 (44.2%) were malignant pathologically. Benign NSNCMs included fibroadenoma (*n* = 155, 63.8%), adenosis (*n* = 60, 24.7%), ductal papilloma (*n* = 20, 8.2%), inflammatory (*n* = 5, 2.1%), cystic ductal dilatation (*n* = 2, 0.8%), and tubular adenoma (*n* = 1, 0.4%). Malignant NSNCMs included invasive ductal carcinoma grade Ⅰ (*n* = 8, 4.2%), invasive ductal carcinoma grade Ⅱ (*n* = 95, 49.5%), invasive ductal carcinoma grade Ⅲ (*n* = 73, 38%), mucinous carcinoma (*n* = 3, 1.6%), solid papillary carcinoma (*n* = 3, 1.6%), encapsulated papillary carcinoma (*n* = 1, 0.5%), medullary carcinoma (*n* = 1, 0.5%), invasive tubulocarcinoma (*n* = 1, 0.5%), and ductal carcinoma *in situ* (*n* = 7, 3.6%).

### Univariate Analysis of Mammographic Tumor Signs and Age

The univariate analysis results of mammographic tumor signs and age between benign and malignant NSNCMs are shown in Table 1. There were significant differences in most of the shape, density, margin, mass size, and age. Among them, the tumor signs with malignant risk were indistinct margin, high density, irregular shape, the elderly, and large masses (*p* < 0.001). Benign NSNCMs were more common with circumscribed or obscured margin, equal or low density, and oval shape and associated with about 40 years of age and smaller masses. The round shape was not statistically significant between the two groups (*p* = 0.959).

**TABLE 1:** Univariate analysis of tumor signs and age between benign and malignant NSNCMs.

Characteristic	Total (*n* = 435)	Benign NSNCM (*n* = 243)	Malignant NSNCM (*n* = 192)	Odds ratio [95% CI]	*p* value
Age (years)^a^	46.1 ± 11.6	41.3 ± 10.1	52.2 ± 10.4	1.1 [1.1, 1.1]	<0.001
Size (mm)^a^	22.6 ± 13.1	20.3 ± 10.2	25.5 ± 15.5	1.0 [1.0, 1.1]	<0.001
Shape					
Round	7 (1.6)	4 (1.7)	3 (1.6)	0.9 [0.2, 4.6]	0.959
Oval	366 (84.1)	226 (93.0)	140 (72.9)	0.2 [0.1, 0.4]	<0.001
Irregular	62 (14.3)	13 (5.3)	49 (25.5)	6.0 [3.2, 11.9]	<0.001
Density					
Low	30 (6.9)	27 (11.1)	3 (1.6)	0.1 [0.03, 0.4]	<0.001
Equal	166 (38.2)	128 (52.7)	38 (19.8)	0.2 [0.1, 0.3]	<0.001
High	239 (54.9)	88 (36.2)	151 (78.6)	6.4 [4.2, 10.0]	<0.001
Margin					
Circumscribed	137 (31.5)	125 (51.5)	12 (6.3)	0.1 [0.03, 0.1]	<0.001
Obscured	51 (11.7)	46 (18.9)	5 (2.6)	0.1 [0.04, 0.3]	<0.001
Indistinct	247 (56.8)	72 (29.6)	175 (91.1)	24.1 [13.9, 43.9]	<0.001

AbrData in parentheses are percentages and data in brackets are 95% confidence intervals.; NSNCM, nonspiculate and noncalcified masses; CI, confidence interval.

a Data are means ± standard deviations.

### Optimal Cut-Off Value of Age and Mass Size

**FIGURE 5:**
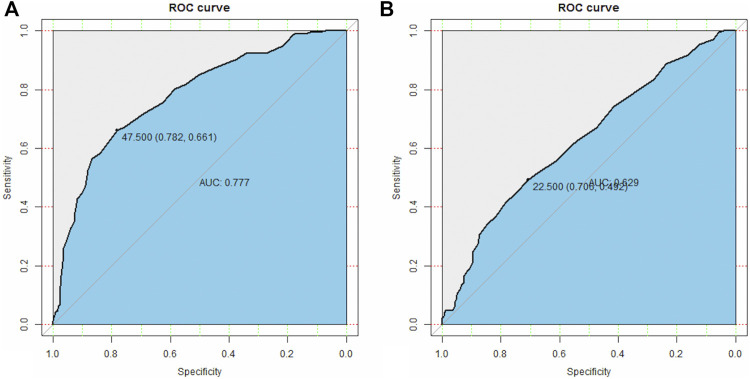
Optimal cut-off value of age and mass size. **(A)** The optimal cut-off of the age is 47.5 **(B)** The optimal cut-off of mass size is 22.5. The ROC analyses were based on continuous data of age and mass size.

The optimal cut-off value of age was 47.5 years by ROC analysis (Figure 5A). The optimal cut-off value of mass size was 22.5 mm by ROC analysis (Figure 5B).

### Univariate Analysis of Mammographic Extratumoral Signs

The univariate analysis results of mammographic extratumoral signs between benign and malignant NSNCMs are shown in Table 2. An overall analysis showed that extratumoral structural abnormalities were highly correlated with malignancy (*p* < 0.001), which appeared externally in 95.3% of malignant masses. In detail, there were significant differences in the subclassification of parenchymal abnormalities. The signs significantly associated with malignancy included parenchymal contraction sign, distortion sign, and atrophy sign, while pushing sign was more common around benign masses (*p* < 0.001). In subclassification of trabecular abnormalities, parallel trabecula sign and vertical trabecula sign were malignant risk factors, and there was significant difference between the two groups (*p* < 0.001), while reticular trabecula sign was not statistically significant (*p =* 0.084). Regarding halo sign, wide halo sign or absent halo were commonly seen in malignancy, while narrow halo was the opposite (*p* < 0.001 to *p* = 0.009).

**TABLE 2:** Univariate analysis of extratumoral signs between benign and malignant NSNCMs.

Characteristic	Total (*n* = 435)	Benign NSNCM (*n* = 243)	Malignant NSNCM (*n* = 192)	Odds ratio [95% CI]	*p* value
ETSA					
Absent	162 (37.2)	153 (63.0)	9 (4.7)	0.03 [0.01, 0.1]	<0.001
Present	273 (62.8)	90 (37.0)	183 (95.3)	33.8 [17.3, 74.3]	<0.001
Parenchyma^a^					
Contraction	58 (13.3)	2 (0.8)	56 (29.2)	45.9 [14.0, 30.4]	<0.001
Distortion	50 (11.5)	4 (1.7)	46 (24.0)	18.1 [7.1, 62.3]	<0.001
Pushing	65 (14.9)	55 (22.6)	10 (5.2)	0.2 [0.1, 0.4]	<0.001
Atrophy	56 (12.9)	13 (5.4)	43 (22.4)	5.1 [2.7, 10.1]	<0.001
Trabecula^a^					
Parallel	153 (35.2)	22 (9.1)	131 (68.2)	21.3 [12.7, 37.1]	<0.001
Vertical	67 (15.4)	11 (4.5)	56 (29.2)	8.6 [4.5, 17.8]	<0.001
Reticular	15 (3.4)	5 (2.1)	10 (5.2)	2.6 [0.9, 8.6]	0.084
Halo					
Absent	214 (49.2)	106 (43.6)	108 (56.2)	1.7 [1.1, 2.4]	0.009
Narrow	110 (25.3)	103 (42.4)	7 (3.7)	0.1 [0.02, 0.1]	<0.001
Wide	111 (25.5)	34 (14.0)	77 (40.1)	4.1 [2.6, 6.6]	<0.001

Data in parentheses are percentages and data in brackets are 95% confidence intervals.

*NSNCM*, nonspiculate and noncalcified masses; *ETSA*, extratumoral structural abnormalities; *CI*, confidence interval.

a Percentage was proportion of each subclassification of parenchymal or trabecular structural abnormalities to the total number, to the benign NSNCM or malignant NSNCM.

### Multivariate Logistic Regression Analysis

Logistic regression analysis results of variables associated with malignant NSNCMs are shown in Table 3. Extratumoral contraction sign of parenchyma was the strongest independent predictor of malignant NSNCM (odds ratio [OR] 36.2, *p* < 0.001), followed by parenchymal distortion (OR 10.2, *p* < 0.001), parallel trabecula sign (OR 7.2, *p* < 0.001), and indistinct margin of tumor (OR 4.3, *p* < 0.001), and also extratumoral atrophy sign of parenchyma (OR 4.0, *p* < 0.001), wide halo (OR 4.0, *p =* 0.022), vertical trabecula sign (OR 3.5, *p* < 0.001), age ≥47.5 years (OR 2.9, *p* < 0.001), irregular shape (OR 2.5, *p =* 0.007), and size ≥22.5 mm of tumor (OR 1.3, *p =* 0.002). Factors not independently associated with malignancy included high density of tumor, extratumoral reticular trabecula, and absent halo (*p* > 0.05).

**TABLE 3:** Multivariate logistic regression analysis of variables associated with malignant NSNCM.

Variable	Odds ratio	95% confidence interval	*p* value
Age ≥ 47.5 years	2.9	1.6, 5.3	<0.001
Size ≥ 22.5 mm	1.3	0.7, 2.5	0.002
Tumor signs			
Irregular shape	2.5	1.0, 6.4	0.007
High density	1.5	0.8, 2.9	0.061
Indistinct margin	4.3	2.1, 9.0	<0.001
Extratumoral signs parenchyma			
Contraction	36.2	11.3, 17.0	<0.001
Distortion	10.2	3.5, 34.3	<0.001
Atrophy	4.0	1.8, 9.4	<0.001
Trabecula			
Parallel	7.2	3.9, 13.7	<0.001
Vertical	3.5	1.5, 8.7	<0.001
Reticular	1.7	0.4, 7.0	0.259
Halo			
Absent	2.5	0.9, 7.2	0.736
Wide	4.0	1.5, 11.9	0.022

*NSNCM*, nonspiculate and noncalcified masses.

### PPV and ROC Curve Analyses of Independent Risk Factors

**FIGURE 6:**
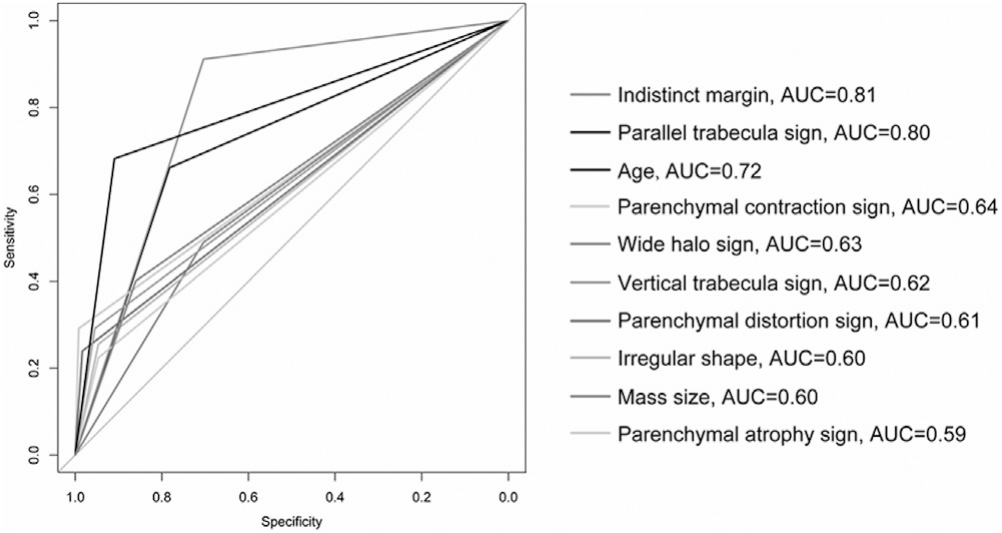
ROC curve of independent malignant risk factors. The optimal cut-off 47.5 was selected using the age prediction model. The optimal cut-off 22.5 was selected using the size prediction model. The figure was edited using Adobe illustrator (version number CS6) (without changing the result).

The results of statistical diagnostic indicators and the ROC curve in evaluating independent malignant risk factors are shown in Table 4 and Figure 6. The PPV and AUC of important independent predictors are shown below: extratumoral contraction sign of parenchyma had the highest PPV (96.6%) and moderate AUC (0.64), parenchymal distortion had higher PPV (92%) and moderate AUC (0.61), parallel trabecula sign also had higher PPV (85.6%) and high AUC (0.80), and indistinct margin of tumor had both high PPV (70.9%) and AUC (0.81). Other predictors had varying PPV (range, 56.6–83.6%) and moderate or near-moderate AUC (range, 0.59-0.72).

**TABLE 4:** Statistical diagnostic indicators of independent malignant risk factors.

	PPV (%)	NPV (%)	Sensitivity (%)	Specificity (%)	Accuracy (%)
Age ≥ 47.5 years	70.6 (127/180)	74.5 (190/255)	66.1 (127/192)	78.2 (190/243)	72.9 (317/435)
Size ≥ 22.5 mm	56.6 (94/166)	63.6 (171/269)	49.0 (94/192)	70.4 (171/243)	60.9 (265/435)
Tumor signs					
Irregular shape	79.0 (49/62)	61.7 (230/373)	25.5 (49/192)	94.7 (230/243)	64.1 (279/435)
Indistinct margin	70.9 (175/247)	91.0 (171/188)	91.1 (175/192)	70.4 (171/243)	79.5 (346/435)
Extratumoral signs parenchyma					
Contraction	96.6 (56/58)	63.9 (241/377)	29.2 (56/192)	99.2 (241/243)	68.3 (297/435)
Distortion	92.0 (46/50)	62.1 (239/385)	24.0 (46/192)	98.4 (239/243)	65.5 (285/435)
Atrophy	76.8 (43/56)	60.7 (230/376)	22.4 (43/192)	94.7 (230/243)	62.8 (273/435)
Trabecula					
Parallel	85.6 (131/153)	78.4 (221/282)	68.2 (131/192)	90.9 (221/243)	80.9 (352/435)
Vertical	83.6 (56/67)	63.0 (232/368)	29.2 (56/192)	95.5 (232/243)	66.2 (288/435)
Wide halo	69.4 (77/111)	64.5 (209/324)	40.1 (77/192)	86.0 (209/243)	65.7 (286/435)

*PPV*, positive predictive value; *NPV*, negative predictive value.

## Discussion

Our study showed that most of the extratumoral signs were independent predictors for malignant NSNCM. Among them, the malignant risk and PPV of subclassification of extratumoral structure abnormalities were higher than tumor signs in different degrees. The diagnostic performance of most of the extratumoral signs was between that of indistinct margin and irregular shape of tumor. This is a gratifying result, which means that for the digital mammographic evaluation of NSNCM, we need to analyze the tumor signs and extratumoral signs at the same time.

In this study of NSNCM, margin and shape of tumor were significantly different from benign to malignant. Consistent with other research studies, an indistinct margin or irregular shape is a suspicious malignant feature (Liberman et al., 1998; Kettritz, 2005; Berment et al., 2014). Since the subject of our study was NSNCMs and extratumoral signs were included, the independent malignant risk of indistinct margin or irregular shape was not higher; it was lower than in previous literatures (Liberman et al., 1998; Kettritz, 2005; Berment et al., 2014). Our data showed that 70.9% of the indistinct margin masses and 79% of the irregular masses were malignant; the PPVs of these were higher than in other literatures (Burrell et al., 1996; Liberman et al., 1998). However, the proportion of irregular shape in malignant NSNCM is not high, but the oval shape was the majority (72.9%) in our study. Then, high density was not independent of malignancy in the study. There are different opinions on the reliability of density in predicting malignancy (Xu et al., 2014; Woods et al., 2021). More attention should be paid to malignant tumors with similar morphological manifestations to those of benign. In this study, 8.8% of NSNCMs with circumscribed margin were malignant, which was similar to a 9% frequency of carcinoma in circumscribed masses reported by Liberman L (Soysal et al., 2015; Dias et al., 2019). Furthermore, circumscribed masses on tomosynthesis images are not guaranteed to be benign lesions (Xu et al., 2014; Woods et al., 2021). Therefore, more morphologic signs were needed to stratify the risk of NSNCM.

Duo to the interaction between the heterogeneity of breast cancer and the organism microenvironment, a variety of growth and spread modes of tumor are determined (Soysal et al., 2015; Dias et al., 2019). Tumors and their surrounding area represent spatially organized “ecosystems” (Sofopoulos et al., 2019). Outward invasion of breast carcinoma and defense response of the organism will inevitably show different signs in different imaging. In order to obtain more information for predicting malignant NSNCM, we classified the extratumoral signs into extratumoral structural abnormalities (parenchymal and trabecular) and halo sign. Owing to the diversity of them, further subclassification was carried out. In this study, masses with extratumoral structural abnormalities were significantly correlated with malignancy. The subclassification sign may appear severally or several may coexist. It indicated that the tumor signs and extratumoral signs of breast carcinoma are an inseparable whole on the image.

The study showed that most of the subclassification of extratumoral structural abnormalities was independently associated with malignancy, which is of positive significance to evaluating NSNCM. Extratumoral contraction sign of parenchyma was the strongest independent predictor of malignancy, followed by parenchymal distortion sign and parallel trabecula sign, the risks of which were higher than indistinct margin of tumor. In addition, the malignant risk of extratumoral parenchymal atrophy sign and vertical trabecula sign was also higher than that of irregular shape of tumor. It indicated that the independent risk factors of extratumoral structural abnormalities were greatly significant for mammographic evaluation of malignant NSNCM compared with the tumor signs.

Among all extratumoral independent predictors for malignancy, parenchymal contraction sign showed the highest PPV (96.6%) and moderate diagnostic performance in our study. Desmoplastic reaction may be a marker of local malignancy (Mezi et al., 1997), and this phenomenon was considered to be a reaction and response of the host tissue against tumor (Martinez and Smith, 2021). Much periductal fibrosiselastic reaction (Uchiyama and Fukuda, 1989; Zhou et al., 2014) may probably be the most direct cause of contraction sign. The sign can be manifested as a banded or “wedge-shaped” contraction of peritumoral or quadrantal parenchyma, also the traction of the edge. Nearly one-third of the malignancies showed parenchymal contraction sign in this study, which is relatively easy to identify on a mammogram. Another independent predictor was the distortion sign of parenchyma with higher PPV (92%) and moderate diagnostic performance, which may be related to desmoplastic reaction or edema (Uematsu, 2015). Parenchymal deformation may appear around the mass or the whole breast. The possibility of extensive edema by lymphatic tumor emboli (Liu et al., 2020) should be considered when the mass is accompanied by diffuse distortion of parenchyma. Furthermore, parenchymal atrophy sign was also an independent predictor with high PPV (76.8%), which may be related to the dominant growth of carcinoma. Atrophic sign may be evaluated by contrasting bilateral breasts because of individual differences.

The invasion of carcinoma and host reaction will not only cause abnormalities in extratumoral parenchyma but also the trabecular structure. Abnormal trabeculae may be hyperplastic fibrous, dilated lymphatic vessels, or ductal system. For subclassification, parallel and vertical trabecula sign were independent predictors for malignant NSNCM, the PPVs of which were 85.6 and 83.6%, respectively. Parallel trabecula sign had high diagnostic performance, which was similar to that of tumor indistinct margin. According to our experience and the study, parallel trabecula sign also has high predictive value for evaluation of malignant NSNCMs, which occur in 68.2% malignancy but only in 9.1% benign masses. Approximately parallel trabeculae surround the mass and are present even away from the mass, and may also appear in deep fat or subcutaneous fat. Extratumoral trabecular abnormalities may exist alone or together with parenchymal abnormalities. Mammography is useful for showing the direction and distribution of trabeculae. Comparative observation or experience is also needed.

Here, we would like to mention the architectural distortion in the BI-RADS lexicon (D’Orsi et al., 2013), which is defined as no visible masses, the appearance of thin straight lines radiating from a point, and focal retraction, distortion, or absence of curvature of the parenchymal edge. In the part of BI-RADS associated features, architectural distortion can be used in combination with other imaging findings to indicate the deformation and retraction of parenchyma near the lesion. Some literatures reported mammographic architectural distortion with different PPVs; however, masses were excluded (Shaheen et al., 2011; Bahl et al., 2015). Biopsy is required even when tomography finds more architectural distortions that reduce the PPV (Alshafeiy et al., 2018). However, detailed analyses of architectural distortion associated with masses are rarely reported. In the study, subclassification of extratumoral signs includes but is not limited to this descriptor. Also, the thin lines from a point are not suitable for masses, while the most common sign around malignant NSNCM is parallel trabecula sign. The extratumoral structure abnormalities may represent different pathologic mechanisms from pure architectural distortion. Therefore, it is necessary to classify and subclassify the extratumoral signs separately in order to supplement predictable information of malignancy on a mammogram.

Regarding extratumoral halo sign, although halo sign is well known for radiologists, there are different reports about its formation. The usual result is from compression of fat by circumscribed mass. Also, study suggested that the halo was a perceptual illusion (Mach band) (Gordenne and Malchair, 1988). Previous literature reported that halo sign could be considered as a marker of benign lesion in females <50 years (Sánchez-Camacho González-Carrato et al., 2018). Our data analysis showed that wide halo sign was associated independently with malignant NSNCM, but the PPV of it was not high compared with those of other extratumoral signs. In addition, two basic pieces of information including age and mass size were statistically analyzed. After univariate analysis showed significant difference, the optimal cut-off values were further determined in order to facilitate the reference in clinical practice. Age ≥47.5 years and mass size ≥22.5 mm were also independent risk factors for malignancy. 70.6% mammographic NSNCMs were malignant in patients older than 47.5 years. In the elderly, those masses that appear to be “benign” are carefully evaluated and further biopsies may be needed.

Our study has several limitations. First, this was a retrospective single-center study. Furthermore, morphological analysis was performed only. The pathological mechanism of extratumoral signs needs to be further explored. The signs were based on visual evaluation and some require experience, so there may be differences between observers. As for the microlobulated margin of mass, it may be more suitable to describe the morphology, so there was no record in our study at present. Also, our study excluded phyllodes tumor because of special biological behavior.

In conclusion, morphological classification and subclassification of extratumoral signs were performed in this study and indicated that the subclassification of extratumoral structural abnormalities have important predictive value for malignant NSNCM on digital mammography. The combination of extratumoral signs identified at mammogram with tumor signs may provide better malignant prediction in patients with NSNCM than tumor signs alone. Whether for prediction of malignancy or further prediction of biological behavior, the extratumoral signs, especially the subclassification of extratumoral structure abnormalities, should be paid continuous attention.

## Data Availability

The raw data supporting the conclusion of this article will be made available by the authors, without undue reservation.

## Abbreviations

AUC, area under the receiver operating characteristic curve; BI-RADS, Breast Imaging Reporting and Data System; CI, confidence interval; ETSA, extratumoral structural abnormalities; NSNCM, nonspiculate and noncalcified masses; NPV, negative predictive value; OR, odds ratio; PACS, picture archiving and communication system; PPV, positive predictive value; ROC, receiver operating characteristic.
